# A Note on a Case of Swallowing False Teeth

**Published:** 1899-07

**Authors:** J. S. Sharman


### A Note on a Case of Swallowing False Teeth.

BY J. S. SHARMAN, L.R.C.P. LOND., M.R.C.S. ENG.

Early on a Sunday morning a lady swallowed three false teeth attached to a gold plate. At one end of the plate was a sharp hook to put round the next tooth and at the other two sharp points; the measurement longitudinally was $1\frac{6}{16}$ in. and transversely $\frac{6}{16}$ in. The patient was awakened by a sense of suffocation. She pressed her throat and she stated that she could feel "them go down." There was practically no discomfort and the teeth were passed on the following Friday morning. On the Thursday the Roentgen rays were used but the teeth could not be detected, although from the back one could plainly see through the body, as was proved by placing a small coin over the region of the abdomen. The operator came to the conclusion that the teeth must be hidden by one of the crests of the ilia, as was no doubt the case.

It is worthy of note that experiments on the cadaver have been of a little use to test the capacity of the stretching of the sphincter of the pylorus. In these experiments a five-franc piece split the sphincter, whereas in the living subject spoons and forks have passed without discomfort. The ileo-cæcal valve and vermiform appendix give more trouble in children than in adults. In most of the text-books very little or nothing is stated besides the various operations as to the treatment of these cases. Rest in the recumbent position and possibly on the right side is desirable, and for diet boiled white of egg, boiled cream, potatoes, rice, and porridge, and if there is any discomfort opium should be administered. In some cases laxatives have been used with success, but this must greatly depend upon the form of the foreign body. In one case where false teeth were swallowed it is stated that oakum, figs, and raisins were given and after a week the body was expelled with the oakum and figs attached to the irregularities of the plate and teeth.—*The Lancet.*

